# Origins of human genetics. A personal perspective

**DOI:** 10.1038/s41431-020-00785-7

**Published:** 2021-02-04

**Authors:** Eberhard Passarge

**Affiliations:** Institut für Humangenetik, Universitätsklinikum Essen, Essen, Germany

**Keywords:** Genetic counselling, Genetics

## Abstract

Genetics evolved as a field of science after 1900 with new theories being derived from experiments obtained in fruit flies, bacteria, and viruses. This personal account suggests that the origins of human genetics can best be traced to the years 1949 to 1959. Several genetic scientific advances in genetics in 1949 yielded results directly relating to humans for the first time, except for a few earlier observations. In 1949 the first textbook of human genetics was published, the American Journal of Human Genetics was founded, and in the previous year the American Society of Human Genetics. In 1940 in Britain a textbook entitled *Introduction to Medical Genetics* served as a foundation for introducing genetic aspects into medicine. The introduction of new methods for analyzing chromosomes and new biochemical assays using cultured cells in 1959 and subsequent years revealed that many human diseases, including cancer, have genetic causes. It became possible to arrive at a precise cause-related genetic diagnosis. As a result the risk of occurrence or re-occurrence of a disease within a family could be assessed correctly. Genetic counseling as a new concept became a basis for improved patient care. Taken together the advances in medically orientated genetic research and patient care since 1949 have resulted in human genetics being both, a basic medical and a basic biological science. Prior to 1949 genetics was not generally viewed in a medical context. Although monogenic human diseases were recognized in 1902, their occurrence and distribution were considered mainly at the population level.

## Introduction

With the completion of the Human Genome Project in 2004 [1] human genetics moved into a new era of exploring the whole genome and its relation to the causes of genetic disorders. New approaches based on numerous new technological advances, such as different automated DNA sequencing methods [2], the elucidation of different types of individual genetic variation [3] and others, allow high resolution analysis of the human genome in various genetic etiologies of diseases [4, 5] in a great number of individuals in different geographic populations [6–9] or analysis of single cells [10]. Earlier genetic studies in human genetics were aimed at individual genes or groups of linked genes. In contrast, during the first 4–5 decades of increasing knowledge of general genetics since 1900, aspects relating to humans could rarely be considered [11–17]. The term “human genetics” has only been in wide use since 1949 on. “Man is one of the most unsatisfactory of all organisms for genetics studies.” One sentence later: “Obviously no geneticist would study such a refractory object, were it not for the importance that a knowledge of the subject has in other fields.” Thus wrote Alfred H Sturtevant in 1954 [18], expressing an opinion widely held among geneticists before the advent of human genetics (Extended Text #1 in Supp. Mat.).

How did human genetics arise? Here I propose that the origins of human genetics as an independent scientific field can best be traced to the years between 1949 and 1959, when genetic advances could be applied to humans. Several scientific events took place in 1949 that support this idea. In addition, I will briefly review advances relating to human genetics as they apply to medicine and patient care before and after 1949, much of it as a personal witness since 1963.

## The year 1949

Two new important insights in 1949 serve as hallmarks in the development of early human genetics. James V Neel described sickle cell anemia as an autosomal recessive trait [19] and four months later in the same volume of *Science* Linus Pauling identified this disorder as a “molecular” disease [20]. In 1949 JBS Haldane estimated the mutation rate in humans based on an analysis of seven human diseases to be about 4 × 10^−5^ [21]. Also in 1949, in a publication entitled “Disease and Evolution” JBS Haldane viewed infectious diseases as having potential as an “agent for natural selection” in man [22].

Another landmark paper in 1949 described the serendipitous discovery of a cytologically visible structure in the nucleus of neurons of female cats, but not in males [23]. Subsequently named Barr body, later X-chromatin, this eventually led to the principle of X-chromosome inactivation [24]. The examples above constitute a shift in the paradigm in scientific progress as postulated by Kuhn [25]. According to this theory science not only progresses as continuous accumulation of knowledge, but also by periods of a new paradigm by asking completely new questions in a new context [26].

For additional reasons the year 1949 can be considered a watershed time point from which modern human genetics developed. In 1949 the American Journal of Human Genetics was established, a year after the founding of the American Society of Human Genetics (ASHG). Curt Stern (1902–1981), one of the leading geneticists between 1923 and 1970, published the first textbook in this field, *Principles of Human Genetics* [27].

The first two meetings of the ASHG took place in September 1948 in Washington, DC, and December 1949 in New York City, both under HJ Muller as president. The title of Muller´s presidential address presented at the second annual meeting of the ASHG in 1949 was “Our Load of Mutations” [28]. This was mainly concerned with the consequences of mutations in humans at the population level.

In 1940 in Britain, a textbook appeared entitled *An Introduction to Medical Genetics* by Fraser Roberts [29]. This was the first textbook on medical genetics, and the only one for many years.

The year 1949 is also noteworthy for human genetics in post-war Germany (Extended Text #2 in Supp. Mat.).

### Early advances

The transition from general genetics to human genetics is characterized by recognizing the medical aspects. Newly discovered chromosome abnormalities, hereditary metabolic defects and molecular technology resulted in defining new human diseases due to different genetic causes. Human genetics includes *medical genetics*, devoted to all of its medical aspects and *clinical genetics*, the practice of diagnosis and management of genetic disorders. McKusick in 1993 stated that clinical genetics originated in 1959 when human cytogenetics and biochemical genetics developed into mainstream subjects of research and its medical applications [30]. The term *genomics*, derived from *genome* (coined by Winkler in 1920), was introduced in 1987 [31]. It relates not only to all genes, but also to the molecules regulating their functions and nuclear structures.

The European Society of Human Genetics (ESHG) was founded at the Third International Congress of Human Genetics in 1966 in Chicago, with the author of this review and Albert de la Chapelle present. Its first annual meeting was held 1968 in Paris.

### Chromosomes

Human genetics is a theory-driven science, but it also greatly depends on advances in methods of investigation. Probably the most important single contribution to the development of modern human genetics was that of cytogenetics in 1959 [32–36]. At first, individual chromosomes in mitosis could not yet be individually identified distinguished except for a few chromosome pairs (Extended Text #3 in Supp. Mat.). New cell culture methods and improved mitotic chromosomal preparations for light microscopic analysis directly led to the recognition in 1959/60 that several human disorders result from defined aberrations in the number or structure of chromosomes (Trisomies 21, 18, 13; partial chromosomal deletions or duplications). Since each aberration was associated with a distinct phenotype, a relationship between a genotype and a phenotype could be defined. In 1959, individuals without a Y chromosome were shown to be female [37], whereas those with a Y chromosome were male no matter how many X chromosomes were present [38]. This was the first step towards defining the fundaments of mammalian sex determination. In the 1960s and 1970s it became apparent that fetal death is frequently caused by chromosomal aberrations that are not observed in newborns. Although chromosomes in metaphase were described as early as 1879, the correct number of human chromosomes was not established until 1956 (Extended Text #4 in Supp. Mat.).

### Cell cultures and biochemical defects

From the 1960s on, cultured cells became widely used to investigate monogenic human diseases (somatic cell genetics). Cells homozygous for a genetic defect could be distinguished from heterozygous cells. Fused homozygous cells from different patients (cell hybrids) could result in a normal cellular phenotype, proving the disease in question to be genetically heterogeneous. Biochemical assays began to define human hereditary metabolic diseases such as amino acid disorders, lysosomal storage diseases, and others at the level of the phenotype and genotype. Prenatal genetic diagnosis was introduced in the late 1960s.

### Molecular advances

Beginning in 1974 DNA could be analyzed by applying new recombinant DNA methods directly, or indirectly by using linked polymorphic DNA markers. New methods to sequence DNA nucleotides in 1977 and to amplify small amounts of DNA in 1985 (PCR) resulted in precise genetic diagnoses with correct assessment of the genetic risk within a given family. Molecular cytogenetics was introduced shortly after 1985. This allowed the analysis of mitotic chromosomes by in situ DNA hybridization. Submicroscopic chromosomal alterations (less than 4 million base pairs of DNA) became visible. New automated massive parallel DNA sequencing methods (“next generation”) introduced in 2005 have made it possible to sequence the DNA of large numbers of individuals and tumor cells at relatively low cost [2, 4]. Other new approaches have become possible: genome-wide association studies (GWAS), exome sequencing, whole genome sequencing, and others.

### Genetics in medicine

From about 1960 on genetics included its medical aspects. McKusick in 1992 reviewed the development of human genetics from the First International Congress of Human Genetics in 1956 at Copenhagen to 1991 [39]. He noted that by 1992 human genetics had become “medicalized, subspecialized, professionalized, molecularized, consumerized, commercialized”. Systematic genetic diagnostic services and genetic counseling became part of patient care [40]. The American Board of Medical Genetics was established in 1979, the American College of Medical Genetics in 1992.

Details of the early stages of developing human genetics are reviewed by McKusick [40], Polani [41], Harper [42, 43], Harper et al. [44]; McKusick & Harper and Childs & Pyeritz [45, 46], and more recently Clausnitzer et al. [4]. Childs in 1999 and 2013 [47, 48] has drawn attention to two views of disease: the classification of diseases differs in medicine and medical human genetics. In medicine it is mainly based on the phenotype, i.e., clinical manifestation, whereas the genetic classification system is based on the genotype, i.e., different types of mutations or other structural rearrangements. Table 1 lists the main genetic features of genetic disorders first described by their phenotypes since 1949. It is remarkable that many of these recognizable phenotypes were not described earlier, such as, e.g., trisomy 18, whereas the phenotype of trisomy 13 was described in 1657 (Thomas Bartholin, “Monstrum sine oculis”). Most disorders listed in Table 1 can be classified according to their genotypes rather than their phenotypes. Their classification is based on different pathogenic causes, such as impaired functions in genome structure, chromatin regulation, cell receptors, transcription factors, signaling pathways, imprinting, and others (for other examples of genetic classification of diseases see Extended Text #5 in Supp. Mat.).

**Table 1:** Examples of new genetic disorders described 1949–2009.

Year	Disorder	OMIM	Genes involved
1952	X-linked agammglobulinemia type Bruton	300300	BTK at Xq22.1, a B-cell regulator
	Lowe syndrome (Inositol phosphate metabolism)	309000	OCRL (300535) at Xq26.1
1954	Bloom syndrome (DNA helicase disorder)	210900	RECQL3 (604610) at 15q26.1
	Russell-Silver syndr. (Heterogenous imprinting disorder)	180860	Hypomethylation IC 11p15.5; 3 other chromosomes
1956	Prader-Willi syndrome (imprinting defects)	176270	SNRPN, NDN; others at 15q11.2
1958	Ataxia telangiectatasia (ATT; Louis-Bar; see NBS 1981)	208900	ATM (601232) at 11q22.3
1959	Alström syndrome (Ciliopathy, centriole function)	203800	ALMS1 (606844) at 2p13.1
1960	Trisomy 18, Trisomy 13	None	New malformation syndromes
1961/62	Williams-Beuren syndrome (contiguous gene syndrome)	194050	homozygous del 7q11.23 of 1.5-1.8 Mb
1962	Menkes disease (Cu transport metabolism)	309400	ATP7A (300011) at Xq21.1
1963	Deletion 5p (Cri-du-chat syndrome)	123450	Terminal deletions at 5p15.2-p15.3
	Rubinstein-Taybi syndr (transcriptional coactivator defect)	180849	del 16p13.3 (CREBBP 600140); 22q13.2 EP300 (602700)
	Miller-Dieker (lissencephaly complex)	247200	Deletions at 17p13.3 and others, involving 11 genes
1964	Deletion 4p (Wolf-Hirschhorn syndrome)	194190	Terminal deletions at 4p16.3
	Smith-Lemli-Opitz syndrome (Cholesterol metabolism)	270400	DHCR7 (602858) at 11q13.4
	Lesch-Nyhan syndrome (Purin metabolism)	300322	HPRT1 (308000) at Xq26.2-q26.3
	Sotos syndrome (see Weaver syndr. 1974)	117550	NSD1 (606681) at 5q35; APC2; NFIX
	Pfeiffer syndr.(Fibrobast growth factor receptor defects)	101600	FGFR1 at 8p11.23; FGFR2 at 10q26.13
	Alagille (2 types; disrupted NOTCH signaling pathway)	118450	JAG1 at 20p12.2; NOTCH2 at 1p12
1965	Angelman syndrome (Imprinting defects)	105830	UBE3A at 15q11.2
1966	Langer-Giedion syndr. (Trichorhinophangeal syndrome)	190350	TRPS1 (604386) at 8q23.3 (Zn-finger transcription factor)
1967	I-cell disease (Mucolipidosis II; lysosomal storage)	252500	GNPTAB (607840) at 12q23.2
	Thanatophoric dysplasias (FGF receptor defects)	187600	FGFR3 (134934) at 4p16.3
	Zellweger syndrome (Peroxisome disorders)	214100	Mutations in PEX genes at 13 loci
1968	Xeroderma pigmentosum (DNA repair disorders)	278700	XPA-XPG (7 autosomal loci, one X-linked XPV)
	Noonan syndrome (RAS-MAPK signaling defects)	163950	PTPN11 (176876) and 12 other genes
	DiGeorge syndrome (see Shprintzen syndr. 1981)	188400	TBX1 haploinsufficiency del at 22q11.2
1969	Fragile X-syndrome (Martin-Bell syndr.)	300624	FMR1, CGG repeat expansion
	Beckwith-Wiedemann syndrome (Imprintig defects)	130650	Mutat. or del of one of four imprinted genes at 11p15.5
	EEC syndrome (one of 6 phenotypes involving TP63)	129900	TP63 (603273) regulator gene at 3q28
	LEOPARD syndrome (3 types)	151100	PTPN11 (176876), RAF1 (611664), BRAF
	Joubert syndrome type 1 (Ciliopathies)	213300	16 autosomal genes, 1 X-linked
1970	Coffin-Siris syndrome (Chromatin dysregulations)	135900	ARID1A (614556) and 10 other genes
1972	Townes-Brocks syndrome (3 genetic types)	107480	SALL1 transcription factor at 16q12.1; DACT1 (607861)
1974	Weaver syndr. (see Sotos 1964 (Chromatin disorders)	277590	EZH2 (601573) at 7q36.1 (Nucleosome histone H3 function
1975	Antley-Bixler syndrome (2 types)	201750	POR (124015) P450 oxidoreductase in type 1; FGFR2 type 2
1977/79	Wiedemann-Rautenstrauch syndr. (Progeroid disorder)	264090	POLR3A (614258) RNA polymerase III
	Costello syndrome (RAS-MAPK signaling pathway)	218040	HRAS at 11p15.5 (see Noonan 1968)
1981	Nijmegen breakage syndrome (see ATT 1958)	251260	NBN (602667) at 8q21.3
	Shprintzen syndrome (Velocardiofacial)	192430	del 1.5-3.0 Mb at 22q11 (see DiGeorge 1968)
	Kabuki make-up syndr. (Chromatin regulatory disorders)	147920	Two types: KMT2D at 12q13.12; KDM6A at Xp11.3
1986	Smith-Magenis syndrome (see Potocki-Lupski 2000)	182290	del 17p11.2, gene RAI1 (607642)
	Cardio-facio-cutaneous syndr. (RAS-MAPK pathway)	115150	BRAF (164757) at 7q34
1993	Nicolaides-Baraitser syndr. (Chromatin dysregulation)	601358	SMARCA2 (600014) at 9q24.3
1997	Muenke syndrome (Fibroblast growth factor receptor)	602849	FGFR3 (134934) at 4p16.3
2000	Potocki-Lupski syndrome (see Smith-Magenis 1986)	610883	dup 17.p11.2
2006	Loeys-Dietz syndrome (Transforming growth factor)	609192	TGFBR1 (190181) at 9q22.33
2009	Kleefstra syndr. (phenotypic series with about 50 members)	610253	EHMT1 (Eurchromatic histone methyltransferase)
	Data based on OMIM [49]. This list is not complete.		
	The new microdeletion/microduplication syndromes since about 1985 are not included		

Table S1 lists examples of major advances in human genetics between 1949 and 2020. The criteria for selection are based on how each entry has been perceived in the literature and personal observations since 1963. The left column contains advances directly relating to human genetics, and the right column entries indirectly contributing to human genetics.

Nowhere is the enormous progress in the medical aspects of human genetics (*medical genetics*), in particular for monogenic disorders, more visible than in *Mendelian Inheritance in Man. A Catalog of Human Genes and Genetic Disorders* (Fig. S1). This was first established in 1966 by Victor A McKusick (1921–2008) at Johns Hopkins University in Baltimore and went through 12 printed editions (1966–1998). Since then it is maintained online as *Online Mendelian Inheritance in Man* (Ref. [49], online freely available at OMIM: www.ncbi.nlm.nih.gov/omim). CF Fraser and H Harris in 1956 independently established genetic heterogeneity as a basic principle in medical genetics [50–53]. Scriver in 1999 [54] first demonstrated that modifying genes influence the phenotype, severity and course of illness in monogenic disorders [55–57]. An important shift of paradigm in genetics occurred when the concept of genetic counseling was introduced (Extended Text #6 in Supp. Mat.).

### Advances in general genetics applied to humans prior to 1949

Prior to 1949 none of the many discoveries in genetics could be derived from direct observations in humans. Advances in genetics generally were not seen in a medical context with patient care. Knowledge of human genetic disorders was aimed at the population level rather than individually to patients and their families. Monogenic Mendelian disorders were viewed as being too rare to be relevant for medical applications and patient care. Complex disorders with multifactorial etiologies had not yet revealed their genetic components. Several of the early genetic investigations in humans were directed at the genetics of normal traits such as stature, color of the eye, skin, hair, mental abilities and the like. They came to erroneous conclusions because the underlying genetic properties are not as simple as assumed at the time. Several presidents of the American Society of Human Genetics and others have reflected on the status of human genetics before 1949 (Extended Text #7 in Supp. Mat.).

A few earlier attempts related genetic knowledge to humans. Neel in 1939 initiated a seminar on human genetics together with Curt Stern (Extended Text #8 in Supp. Mat.). In 1940 in Britain, a textbook appeared entitled *An Introduction to Medical Genetics* by Fraser Roberts [29]. This was the first textbook on medical genetics, and the only one for many years (Extended Text #9 in Supp. Mat.). In Germany in 1923 a 500-page textbook entitled “Human Heredity Science and Racial Hygiene” went through five editions until 1940 (Extended Text #10 in Supp. Mat.).

In 1934 A Følling described phenylketonuria (OMIM 261600) as a cause of mental retardation. After GA Jervis recognized the enzyme defect in 1947, and H Bickel in 1953 delineated an approach to dietary therapy, R Guthrie in 1962 set the stage for population-wide screening of newborns for early diagnosis and effective therapy. Today a great number of hereditary metabolic disorders can be identified in newborns prior to clinical manifestation.

In general however, advances in genetics were not considered in relation to medicine. This would have required a shift in paradigm, which did not occur at that time. A gross misconception in applying genetic considerations to humans in the 1920s and 1930s was *Eugenics* (Extended Text #11 in Supp. Mat.).

### Prescient insights

Three remarkable exceptions with early genetic insights relating to humans can be cited here: William Bateson, Archibald E Garrod, and Theodor Boveri. They can be considered forerunners of human genetics. *William Bateson* (1861–1926) at Cambridge in his *Principles of Heredity* in 1913 [12] described several human pedigrees with autosomal dominant, recessive, and X-linked inheritance (pp. 203–234). Bateson states on page 233: “Similarly when we find that a condition such as retinitis pigmentosa sometimes descends in one way and sometimes in another, we may perhaps expect that a fuller knowledge of facts would show that more than one pathological state may be included under the same name” [12]. Thus, Bateson recognized genetic heterogeneity more than 40 years before CF Fraser and H Harris in 1956 independently established it as a basic principle in human genetics (see above). Other examples of early descriptions of Mendelian inheritance of human diseases are heritable biochemical defects, described by Archibald Garrod as “inborn errors of metabolism” [58–60] or brachydactyly type A1 (OMIM 112500) by WC Farabee in a PhD thesis published in 1905, reviewed by Haws & McKusick in 1963 [61] and Bateson, 1913, page 210–216 [12].

Archibald E Garrod (1857–1936) at Great Ormand Street Hospital London recognized the genetic individuality of man. In a letter to Bateson on 11 January 1902, Garrod wrote: “I believe that no two individuals are exactly alike chemically any more than structurally (Ref. [60], Bearn, 1993, page 61). In his prescient monograph *Inborn Factors of Disease* of 1931 Garrod considered predisposition to disease to be important [47, 48, 60]. A remarkable insight pointing to the importance of genetics in human diseases is contained in Thomas H Morgan’s Nobel lecture in 1934, *The relation of genetics to physiology and medicine*: “… considering the present attitude of medicine and the dominating place of the constitutional researches, the role of the inner, hereditary factors to health and disease appears in a still clearer light. For the general understanding of maladies, for prophylactic medicine, and for the treatment of diseases, hereditary research thus gains still greater importance” (cited by Bearn, 1993, ref. [60], page 193).

The third example is *Theodor Boveri* (1862–1915) at Würzburg. By 1902 he had recognized the individuality of chromosomes [62]. Subsequently Boveri related changes in chromosomes to the causes of cancer [63, 64]. However, more than four decades went by until 1960 when the Philadelphia chromosome was described in chronic myelogenous leukemia [65, 66]. The “One Gene – One Enzyme” hypothesis proposed by George W Beadle in 1941 could have become a corner stone of human biochemical genetics. Beadle referred to Garrod in his Nobel lecture in 1958 (cited by Bearn, 1993, ref. [60], page 150).

### Diversity of modern human genetics

Modern human genetics has evolved in different directions mainly based on different methods of investigation, although in research it is by no means limited to *Homo sapiens*. Today it comprises genomics with several subsections (e.g., proteomics, epigenomics and others), molecular genetics, tumor genetics and -genomics, pharmacogenetics and -genomics, immunogenetics, epigenetics, cytogenetics, somatic cell genetics, biochemical genetics, population genetics, evolutionary bases of causes of disorders, bioinformatics and others. This is extensively reviewed in two current multivolume online textbooks [67, 68]. No vertebrate genetics or genomics is better understood than that of man. Yet, human genetics is not an established curriculum of study within the faculties of either medicine or biology. Rather, to become a human geneticist one must study medicine or a basic science and complete approximately five years of formal postgraduate training. Thus, human geneticists represent either a medical or a non-medical basic science. This dual structure of being both a medical and a biological discipline makes human genetics unique among the medical subspecialities, as outlined in detail by Childs [47, 48].

## Conclusion

In summary, modern human genetics began when new advances in genetics were systematically applied in medicine from 1949 on. A close relationship between genetics and medicine evolved into human genetics. This contributes greatly to an understanding of the causes of human diseases. In addition, genetic counseling based on empathy and free decision-making of individuals has become part of patient care. Human genetics had become “medicalized” [40].

## Supplementary information

Supplementary Material Online

Fig. S1 (in Supplementary Material)

Table S1 (in Supplementary Material)
