# Supplementary material for: Origins of human genetics. A personal perspective
Source: Eur J Hum Genet. 2021 Feb 4;29(7):1038–44. doi: 10.1038/s41431-020-00785-7 (PMC8298510; doi:10.1038/s41431-020-00785-7)
Supplement: Supplementary file 1 — Supplementary Material Online [file 41431_2020_785_MOESM1_ESM.docx]

(MS 840-19-EJHGR. Resubmission #2 2020)

Viewpoint Article

**Supplementary Material Online**

1) Fig. S1. Twelve printed editions of VA McKusick, *Mendelian Inheritance of Man*, 1966-1998 (Photograph of the books courtesy of the late Dr. Victor A McKusick).

2) Table S1. Major advances relating to human genetics.

The criteria for selection are based on how each entry has been perceived in the literature and personal observations since 1963. The left column contains advances directly relating to human genetics and the right column entries indirectly contributing to human genetics.

3) Extended Text (#1 - #11)

4) References to Extended Text

Extended Text #1

Actually Sturtevant was concerned with the genetic effects of radiation. He also wrote: “Another thing that must be avoided is the view that one race (usually that to which one himself belongs) is “better” than another” (1). Alfred H Sturtevant (1891-1970) was one of the leading geneticists in the newly formed *Drosophila* genetics group of Thomas Hunt Morgan (1890-1967) at Columbia University. He joined this group in 1910 in the “Fly Room” at Columbia University (2). In 1913 he established the first genetic map by demonstrating a linear order of six genes (“factors”) on the *Drosophila* X-chromosome (3).

Extended Text #2

In Germany in 1949 Friedrich Vogel (1925-2006), later a leading human geneticist in post-war Germany and chairman of the Institute of Human Genetics, University of Heidelberg 1962-1993, decided to become a human geneticist (F. Vogel, personal communication 6 May 1998). I consider this to have been a rather bold move in a post-war Germany that was devastated and discredited by “eugenics” and “racial hygiene” (see Extended Text #11). After studying medicine, Vogel began to investigate human mutations in 1953, in particular retinoblastoma, at a Max-Planck Institute in Berlin-Dahlem. The grant application on his behalf was the first dealing with genetics in Germany after 1945 (4). Vogel´s *Lehrbuch der Humangenetik* of 1961 was the first textbook on human genetics in Germany after World War II.

Extended Text #3

Several conferences between 1960 and 1971 established criteria for the systematic pairwise arrangement of human chromosomes, the karyogram. After 1970 several different staining methods for mitotic chromosomal preparations were introduced. From then on each chromosome pair could be identified by its specific pattern of intensely or less intensely stained bands along each chromosome. Different types of banding patterns defined chromosomal regions. A system of numbered bands was assigned to each chromosomal region. As a result, the loss (deletion) or duplication of a part of a chromosome could be recognized within the limits of light microscopic resolution. The banding patterns also paved the way to mapping genes to their locations on a specific site of a chromosome.

Extended Text #4

The correct number of human chromosomes was established in 1956 (5, 6). Prior to 1956, the chromosome number had been determined to be 48 by Painter in 1923 and subsequent years (7). At least 40 studies between 1923 and 1956 stated the diploid human chromosome number as 48 rather than the correct number of 46 (8). Observations by Hughes in 1952 yielded 46 chromosomes instead of 48 (9), confirmed by Hsu (10), but the correct number of chromosomes was still not recognized against the preconception of 48 (11). Technically it would have been possible to establish the correct number of human chromosomes and observe human chromosomal aberrations several decades earlier. But this would have required a shift in paradigm according to the theory by Kuhn in 1962 (12), which did not occur at that time.

Extended Text #5

Illustrated examples are: genomic disorders, dysregulated chromatin structure, disorders resulting from rearrangements of *cis*-regulatory elements, defects in telomeres, defective lamins, dysfunctional cohesion (cohesinopathies), dysfunctional cilia (ciliopathies), neural crest disorders (neurocristopathies), dysregulated RAS-MAPK signaling pathway, unstable repeat disorders, and imprinting disorders (Ref 13, pp. 236-259).

Extended Text #6

John Alexander Fraser Roberts (1899-1987) established in Britain the foundations of genetic counseling. In the first edition of his book *An Introduction to Medical Genetics* in 1940 (14, see Extended Text #9) in a section “The attitude of the patient to hereditary abnormalities and disease” he stated that genetic counseling could dispel unfounded fears and explain genetic risks. Fraser Roberts set up a clinic for genetic counseling in London in 1940, the first in Europe (15).

Sheldon Reed, president of the ASHG in 1956, introduced the concept of genetic counseling in the USA (16-18). Sheldon Reed stated: “The primary function of genetic counseling is to provide people with an understanding of the genetic problems in their family”. Paul in 1997 (19) cited Reed as considering genetic counseling as “a kind of genetic social work without eugenic connotations.” Genetic counseling subsequently became a central activity in medical human genetics (20-30). A definition of genetic counseling by the ASHG in 1972 (31) was modified by the National Society of Genetic Counselors (NSGC) in 2005 to read: “Genetic counseling is the process of helping people understand and adapt to the medical, psychological, and familial implications of genetic contributions to disease. This process integrates (1) interpretation of family and medical histories to access the chance of disease occurrence or recurrence, (2) education about inheritance, testing, management, prevention, resources, and research, (3) counseling to promote informed choices and adaptation to the risk or condition” (32). Genetic counseling clearly exceeds the usual medical advice given to a patient during the course of illness. It involves unaffected family members, called “consultants”. It does not provide a decision. Now that direct DNA analysis has became part of diagnostic procedures, some diseases can be diagnosed by predictive genetic testing long before they become manifest (33). This widens the time frame of diagnosis and extends the scope of genetic counseling.

Extended Text #7

In his presidential address at the 1961 ASHG meeting LC Dunn (34) raised the question of why human genetics developed so slowly in the sixty years between 1900 and 1960. This is surprising, indeed, since many basic principles of genetics were known much earlier, except for DNA and the genetic code. Neel (35) viewed human genetics in the 1940s as a two-tier system, biochemical genetics and population genetics. Hirschhorn in 1996 emphasized new concepts such as genetic counseling, birth defects, and prenatal diagnosis (36). He considered human genetics at risk of fragmentation into different directions and by the formation of several new organizations.

Extended Text #8

Curt Stern (1902-1981) was one of the leading geneticists between 1923 and 1970 (37). Neel in 1994 (38) described on page 9 that in 1939, Stern was able to locate no more than two dozen papers dealing with specific inherited human diseases. By about 1941 Neel considered pursuing a career in medical genetics. He called it “a gamble” to devote his professional career to studies of human genetics at a time when most colleagues found this to be impossible and not worth the effort (Ref. 38, p. 16). Neel envisioned introducing the rigor of *Drosophila* genetics into human genetics. But such a turn did not occur before 1949.

Extended Text #9

John Alexander Fraser Roberts (1899-1987) was a leading British geneticist who had developed an early interest in bringing genetic ideas into medicine (14, 15). His book *Introduction to Medical Genetics* went through 7 editions with additional authors until 1978. In the preface to the first edition in 1940, Fraser Roberts stated “that neglect of established principles of medical teaching has been largely responsible for the failure to assimilate and use genetic advances. A textbook of genetics is no more a substitute for one on medical genetics than is a textbook of physiology a substitute for one on medicine" (39). In the preface to the second edition 1959 Fraser Roberts stated that the plan fort the first edition began in 1935 when a medical student (the future Dr. Duncan Duthie) asked for an explanation of genetics. Subsequently Fraser Roberts developed a short course of lectures for medical students. In 1946 Fraser Roberts was appointed a consultant in medical genetics at the Royal Eastern Counties Hospital, Colchester, and started a genetic counseling clinic there until retirement in 1981 (41).

Extended Text #10

This 500-page textbook entitled “Human Heredity Science and Racial Hygiene” first appeared in 1923 (42). It went through five editions until 1940. Today it sometimes still is cited as an early introduction to the medical aspects of genetics. McKusick and Harper (43) credit this publication with having recognized genetic heterogeneity. I find no evidence for this in the text. On 143 pages in the first edition of 1923 the authors cover 15 different human disease states, beginning with the eye (13 pages), hearing (3 pages) and other organ systems or functional areas. The presentations rest mainly on Mendelian pedigrees. The phenotypic descriptions are vague. For several of the disorders mentioned the genetic basis was obscure. Not one word about the impact of the occurrence of a genetic disease on the affected individuals and their families. In my opinion this publication cannot qualify as a forerunner of human genetics inasmuch as it uses the term “racial hygiene” in its title (see Comment #11).

Extended Text #11

Misleading concepts and erroneous genetic conclusions characterize the eugenic movement in the 1920s and 1930s (44-47). Francis Galton (1822-1911) introduced the term *Eugenics* in 1883 as “the study of the agencies under social control that may improve or impair the racial qualities of future generations, either physically or mentally” (48).

CB Davenport (1866-1944) became the chief advocate in America in 1911, defining eugenics as “the science of the improvement of the human race by better breeding”. It was believed that the “white race” was superior to others. The proponents did not realize that genetically defined human races do not exist (49). They assumed that one could reduce the frequency of genetic diseases or eliminate them from the population. In many countries in Europe and the United States in the late 1920s ideas developed on how to “improve” the genetic composition of a population (i.e., to clear it of genetic diseases) by “eugenics” (44, 48). In the United States a “Eugenics Record Office” (ERO) was founded in 1910 at Cold Spring Harbor, Long Island. At first involved in defined human diseases, it soon became occupied with vague and poorly understood traits (Ref. 38, p. 15). In a simplistic approach, genetic and environmental contributions to a trait studied were not distinguished and erroneous conclusions were drawn.

Prominent members of the ERO were enthusiastic about the infamous law of 14 July 1933 in Nazi Germany entitled “Law for the Prevention of Genetically Diseased Offspring” (Gesetz zur Verhütung erbkranken Nachwuchs. Reichsgesetzblatt Nr. 80, 1933, page 529) viewing it as important advancement in “racial hygiene” (50). This “law” mentioned eight “genetic diseases”, such as “mental impairment”, schizophrenia, hereditary blindness, “severe hereditary malformation” etc. None of these are associated with a high genetic risk to offspring. Only one is heritable, Huntington´s chorea. By 1935, sterilization laws had been passed in Denmark, Norway, Sweden, Germany, and Switzerland, as well as in 27 states of the United States (44, 50). Although in most cases the stated purpose was eugenic, sterilizations were performed for social reasons. In Nazi Germany under the totally erroneous eugenic pretext called “racial hygiene”, millions of innocent human beings claimed to be “worthless” were murdered (51).

**References to Extended Text in Supplementary Material Online**

1. Sturtevant AH: Social implications of the genetics of man. Science 1954;120: 405-407.70.

2. Sturtevant AH. *A History of Genetics*. Harper & Row, New York, 1965 (reprinted by Cold Spring Harbor Laboratory Press, 2001).

3. Sturtevant AH. The linear arrangement of six sex-linked factors in Drosophila, as shown by their mode of association. J Exp Zool 1913;14:43-59.

4. Vogel F. Die Entwicklung der Humangenetik in Deutschland nach dem Zweiten Weltkrieg. MedGenet 1999;11:409-418.

5. Tijo JH, Levan A. The chromosome number of man. Hereditas 1956;42:1-6.

6. Ford CE, Hamerton JL. The chromosomes of man. Nature 1956;178:1020-1023.

7. Painter T S. Studies in mammalian spermatogenesis. II. The spermatogenesis of man. J Exp Zool 1923;37:291–336.

8. Ferguson-Smith MA. History and evolution of cytogenetics. Mol Cytogenet 2015 Mar 20; 8:19 (doi: 10.1186/s13039-015-0125-8).

9. Hughes A: Some effects of abnormal tonicity on dividing cells in chick tissue cultures. J Cell Sci 1952;93:207-220.

10. Hsu TC. *Human and Mammalian Cytogenetics. An Historical Perspective*. Springer, Heidelberg-New York, 1979.

11. Kottler MJ. Cytological technique, preconception, and counting of the human chromosomes. Bull Hist Med 1974;48(4):465-502.

12. Kuhn TS: *The Structure of Scientific Revolutions*. University of Chicago Press, Chicago, 1962 (2^nd^ ed 1970, 3^rd^ 1996, 4^th^ ed 2012).

13. Passarge E. *Color Atlas of Genetics.* 5th ed. Thieme Medical Publishers, Stuttgart-New York, 2018.

14. Fraser Roberts JA. *An Introduction to Medical Genetics*. Oxford University Press, Oxford, 1940 (with several subsequent editions with co-authors, 7^th^ in 1978).

15. Pembrey ME. Dr. John Alexander Fraser Roberts. Obituary. J Med Genet 1987;24(7):442-444.

# 16. Reed SC. Hereditary counseling. Eugen Quart 1954;1:48-49.

17. Reed SC. A short history of genetic counseling. Soc Biol 1974;21:332-339.

18. Reed S. *Counseling in Medical Genetics*. Third Edition. Allan R Liss, New York, 1980 (First edition 1955, 2^nd^ ed. 1963).

19. Paul D. From eugenics to medical genetics. J Hist Policy Hist 1997;9(1):96-116.

20. Carter CO, Roberts JAF, Evans KA, Buck AR. Genetic clinic, a follow-up. Lancet 1971;1:281-285.

21. Leonard CO, Chase GA, Childs B. Genetic counseling: a consumers view. New Eng J Med 1972;287(9):433-439.

22. Murphy EA, Chase GA. *Principles of Genetic Counseling*. Year Book Medical Publ., Chicago, 1975.

23. Scriver CR, Laberge C, Clow CL, Fraser FC. Genetics and medicine: an evolving relationship. Science 1978; 200:946-952.

24. Passarge E, Vogel, F. The delivery of genetic counseling services in Europe. Hum Genet

1980;56:1-5.

# 25. Bowles Biesecker B, Marteau TM. The future of genetic counselling: an international perspective. Nature Genet 1999;22:133-137.

# 26. Harper P. *Practical Genetic Counseling*. 7^th^ ed. Edward Arnold, London, 2010.

# 27. Holt RL, Trepanier A. Genetic counseling and clinical risk assessment. Chapter 21. In: *Principles and Practice of Medical Genetics*, 6th ed. Elsevier, New York, 2013.

28. Athens BA, Caldwell SL, Umstead KL, Connors PD, Brenna E, Biesecker BB. A systematic review of randomized controlled trials to assess outcomes of genetic counseling. J Genet Couns 2017; 26:902-933.

29. Yeates L, McEwen A, Ingles J. What do we do and how do we do it? Assessing genetic counselling in the modern era. Eur J Hum Genet 2020;28:1137-1138.

30. Voorwinden JS, Plantinga M, Ausems M, Knoers N, Velthuizen M, Birnie E et al. Cognitive and affective outcomes of genetic counseling in the Netherlands at group and individual level: a personalized approach seems necessary. Eur J Hum Genet 2020; 28:1187-1195.

31. Fraser FC. Genetic counseling. Am J Hum Genet 1974;26:636-659.

32. Resta RG: Defining and redefining the scope and goals of genetic counseling. J Med Genet C Semin Med Genet 2006;142C(4):269-275.

33. Evans JP. The complexities of predictive genetic testing.

Brit Med 2001;322(7293):1052-1056.

34. Dunn LC. Cross currents in the history of human genetics.

Am J Hum Genet 1962;14:1-13.

35. Neel JV. Between two worlds. Am J Hum Genet 1966;18:4-20.

36. Hirschhorn K. Human genetics: A discipline at risk for fragmentation. Am J Hum Genet 1996;58:1-6.

37. Neel JV. Curt Stern, August 30, 1902 - October 23, 1981. Biogr Mem Natl Acad Sci 1986;56:443-473.

38. Neel JV. *Physician to the Gene Pool. Genetic Lessons and other Stories*. John Wiley & Sons, New York, 1994.

39. Opitz JM. Book Review. An Introduction to Medical Genetics. Arch Intern Med 1965;113(6):908-910.

40. Fraser FC. Book Review, *An Introduction to Medical Genetics* by JA Fraser Roberts. 2^nd^ ed. Oxford University Press, 1959. Am J Hum Genet 1960; 12(2):228-22).

41. Polani PE. John Alexander Fraser Roberts, 8 September 1899-15 January 1963. Royal Soc 1963.

42. Baur E, Fischer E, Lenz F. *Menschliche Erblichkeitslehre und Rassenhygiene*. JF Lehmann´s Verlag, München, 1923.

43. McKusick VA, Harper PS: History of Medical Genetics. Chapter 1, p. 1-38. In: *Emery and Rimoins Principles and Practice of Medical Genetics*. 6^th^ ed. DL Rimoin, RE Pyeritz, BR Korf, editors. Elsevier, New York, 2013 (E-book only).

44. Harper PS. Human genetics in troubled times and places. Hereditas 2017;155(7):1-14.

45. Petermann HI, Harper PS, Doetz S, editors. *History of Human Genetics: Aspects of its Development and Global Perspectives*. Springer, Heidelberg, 2017.

46. Strong C: Eugenics. pp. 335-340. In: Cooper DV, editor. *Encyclopedia of the Human Genome*. Vol. 2. Nature Publishing Group, London, 2003.

47. Vogel F, Motulsky AG. *Human Genetics: Problems and Approaches*. Springer, Heidelberg-New York, 1997.

48. Allen GE. The eugenics record office at Cold Spring Harbor, 1910-1940: an essay in institutional history. Osiris, 2^nd^ series, 1986; 2:225-264.

49. Yudell M, Roberts D, DeSalle R, Tishkoff S. Science and society: Taking race out of human genetics. Science 2016;351(6273):564-565.

50. Allen GE. “Culling the herd”: eugenics and the conservation movement in the United States, 1900-1940. J Hist Biol 2013;46(1):31-72.

51. Müller-Hill B. *Murderous Science*. Cold Spring Harbor Laboratory Press. Cold Spring Harbor, 1997.

52. King RC, Mulligan PK, Stansfield WD. *A Dictionary of Genetics*. 8th ed. Oxford University Press, Oxford, 2013.
