# Supplementary material for: Origins of human genetics. A personal perspective
Source: Eur J Hum Genet. 2021 Feb 4;29(7):1038–44. doi: 10.1038/s41431-020-00785-7 (PMC8298510; doi:10.1038/s41431-020-00785-7)
Supplement: Supplementary file 2 — Fig. S1 (in Supplementary Material) [file 41431_2020_785_MOESM2_ESM.pptx]

## Slide 1
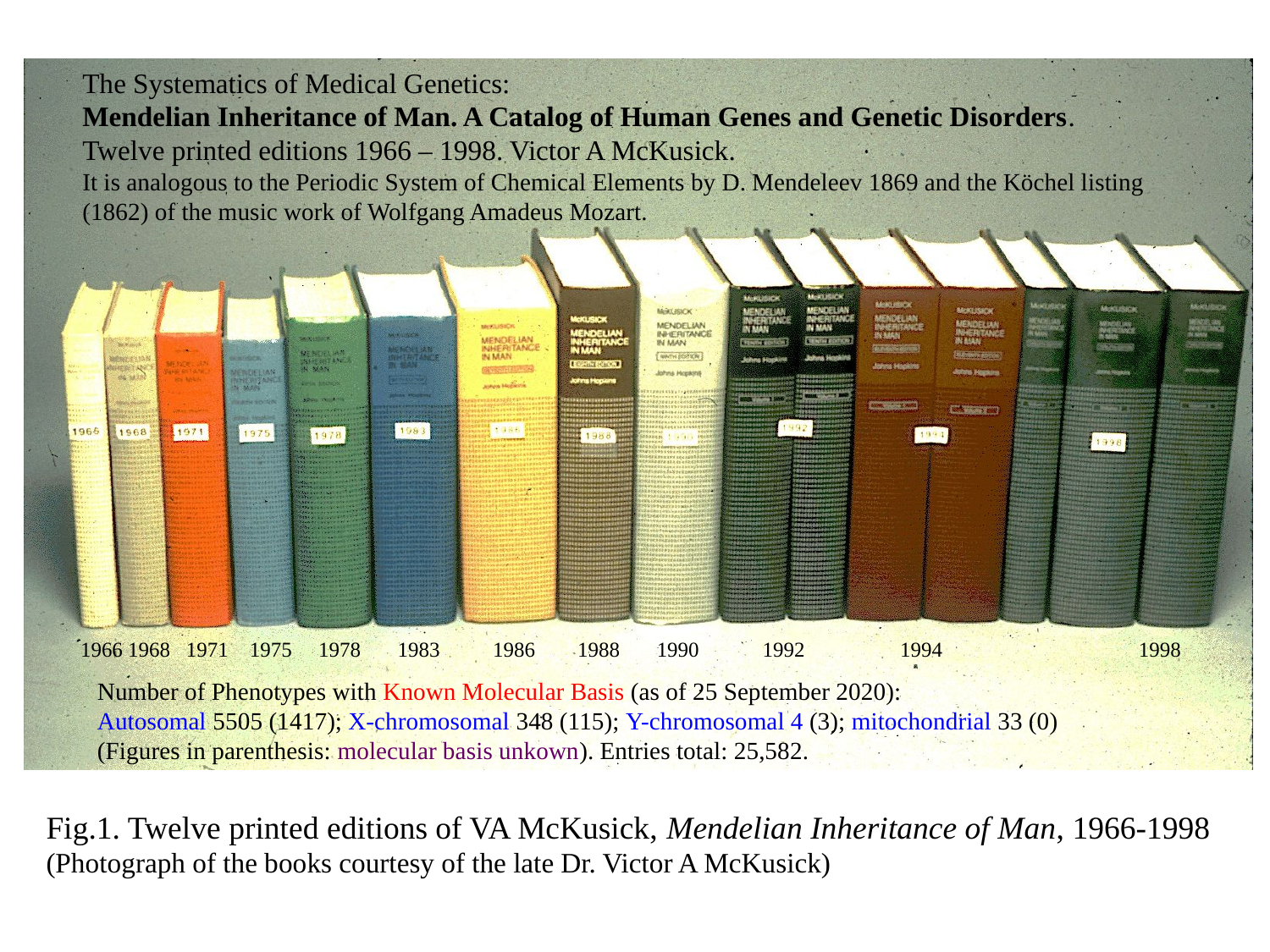

The Systematics of Medical Genetics:
Mendelian Inheritance of Man. A Catalog of Human Genes and Genetic Disorders.
Twelve printed editions 1966 – 1998. Victor A McKusick.
It is analogous to the Periodic System of Chemical Elements by D. Mendeleev 1869 and the Köchel listing (1862) of the music work of Wolfgang Amadeus Mozart.
1966 1968 1971 1975 1978 1983 1986 1988 1990 1992 1994		 1998
Number of Phenotypes with Known Molecular Basis (as of 25 September 2020):
Autosomal 5505 (1417); X-chromosomal 348 (115); Y-chromosomal 4 (3); mitochondrial 33 (0)
(Figures in parenthesis: molecular basis unkown). Entries total: 25,582.
Fig.1. Twelve printed editions of VA McKusick, Mendelian Inheritance of Man, 1966-1998
(Photograph of the books courtesy of the late Dr. Victor A McKusick)

## Slide 2
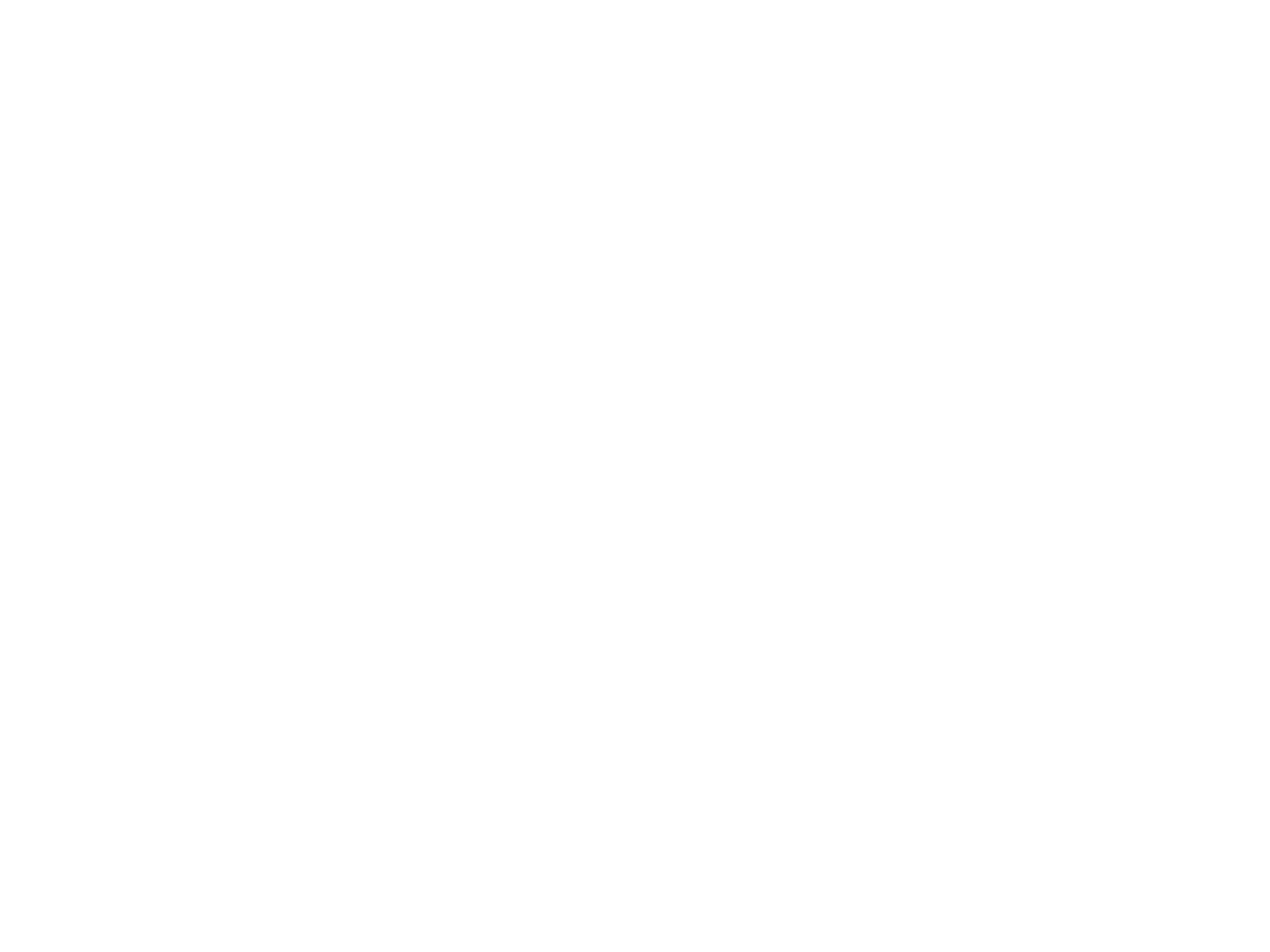

#

## Slide 3
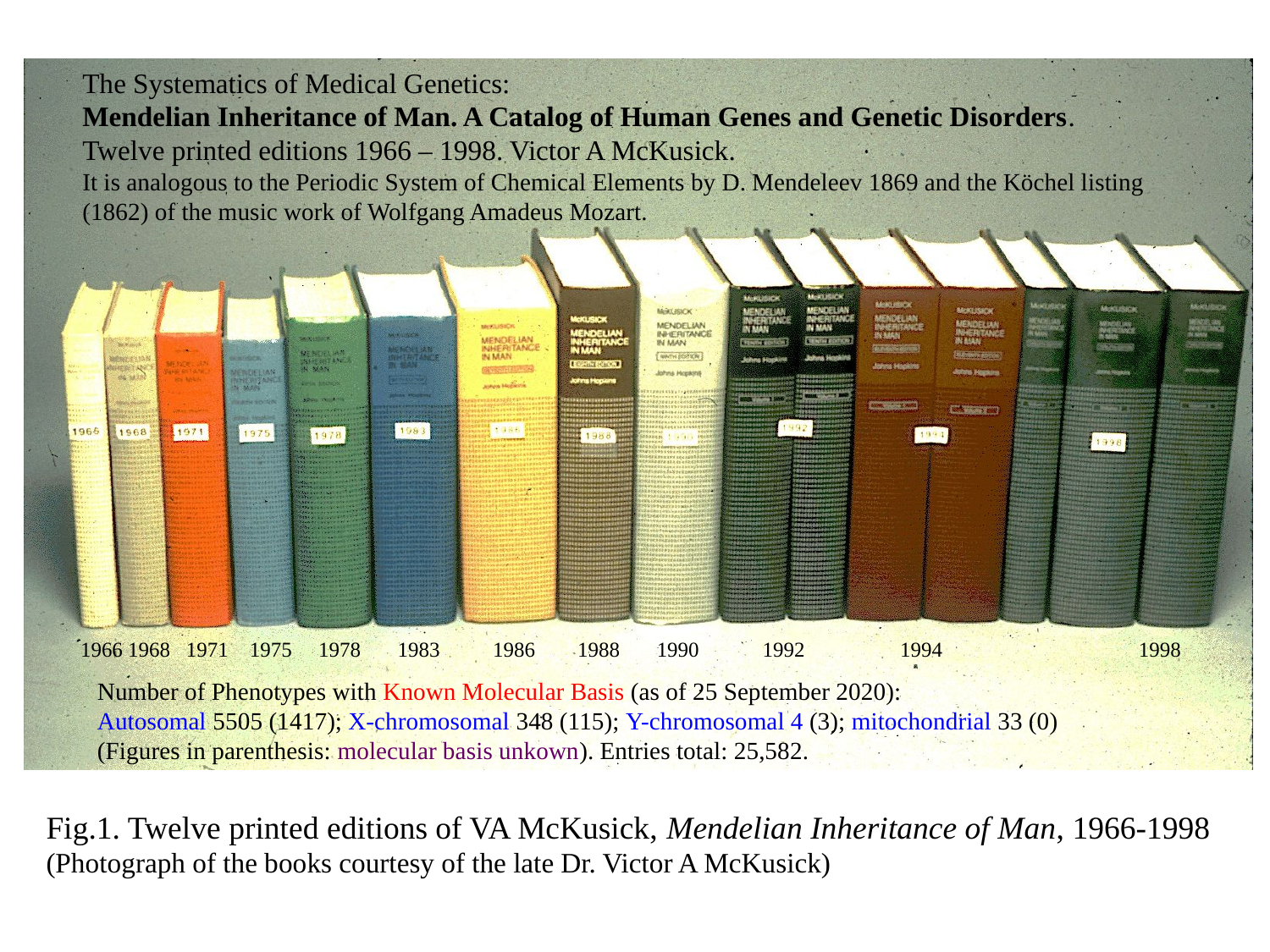

The Systematics of Medical Genetics:
Mendelian Inheritance of Man. A Catalog of Human Genes and Genetic Disorders.
Twelve printed editions 1966 – 1998. Victor A McKusick.
It is analogous to the Periodic System of Chemical Elements by D. Mendeleev 1869 and the Köchel listing (1862) of the music work of Wolfgang Amadeus Mozart.
1966 1968 1971 1975 1978 1983 1986 1988 1990 1992 1994		 1998
Number of Phenotypes with Known Molecular Basis (as of 25 September 2020):
Autosomal 5505 (1417); X-chromosomal 348 (115); Y-chromosomal 4 (3); mitochondrial 33 (0)
(Figures in parenthesis: molecular basis unkown). Entries total: 25,582.
Fig.1. Twelve printed editions of VA McKusick, Mendelian Inheritance of Man, 1966-1998
(Photograph of the books courtesy of the late Dr. Victor A McKusick)
